# Conical petal epidermal cells, regulated by the MYB transcription factor MIXTA, have an ancient origin within the angiosperms

**DOI:** 10.1093/jxb/erac223

**Published:** 2022-05-21

**Authors:** Alison Reed, Paula J Rudall, Samuel F Brockington, Beverley J Glover

**Affiliations:** Department of Plant Sciences, University of Cambridge, Cambridge, UK; Jodrell Laboratory, Royal Botanic Gardens Kew, Richmond, Surrey, UK; Department of Plant Sciences, University of Cambridge, Cambridge, UK; Department of Plant Sciences, University of Cambridge, Cambridge, UK; University of Massachusetts Amherst, USA

**Keywords:** ANA grade, *Cabomba caroliniana*, conical cell, MIXTA, Nymphaeales, papillae, petal, tepal

## Abstract

Conical epidermal cells occur on the tepals (perianth organs, typically petals and/or sepals) of the majority of animal-pollinated angiosperms, where they play both visual and tactile roles in pollinator attraction, providing grip to foraging insects, and enhancing colour, temperature, and hydrophobicity. To explore the evolutionary history of conical epidermal cells in angiosperms, we surveyed the tepal epidermis in representative species of the ANA-grade families, the early-diverging successive sister lineages to all other extant angiosperms, and analysed the function of a candidate regulator of cell outgrowth from *Cabomba caroliniana* (Nymphaeales). We identified conical cells in at least two genera from different families (*Austrobaileya* and *Cabomba*). A single *SBG9 MYB* gene was isolated from *C. caroliniana* and found to induce strong differentiation of cellular outgrowth, including conical cells, when ectopically expressed in *Nicotiana tabacum.* Ontogenetic analysis and quantitative reverse transcription–PCR established that *CcSBG9A1* is spatially and temporally expressed in a profile which correlates with a role in conical cell development. We conclude that conical or subconical cells on perianth organs are ancient within the angiosperms and most probably develop using a common genetic programme initiated by a SBG9 MYB transcription factor.

## Teaser

Some of the earliest diverging flowers have pollinator-attracting conical cells, indicating that the genetic pathway controlling their development is also ancient.

## Introduction

The relationships between flowering plants and their pollinators are key components of ecological networks in almost all terrestrial habitats. Animal pollinators are diverse, with >20 000 pollinating bee species and numerous other insect and vertebrate pollinators (Kevan, 1999). An estimated 35% of global crop production (by volume) depends on biotic pollination, and a decrease in pollinator numbers worldwide has led to a reduction in some crop production rates (Klein *et al.*, 2007). The evolutionary radiation of angiosperms and their insect pollinators has resulted in considerable diversity of floral forms, with a range of floral traits thought to have evolved in response to selective pressure to increase floral attractiveness and memorability (Kevan and Baker, 1983; Schiestl and Johnson, 2013). These traits include flower scent and reward, as well as visual cues involving colour, shape, and patterning.

In the majority of biotically pollinated angiosperm species, the perianth organs involved in visual advertising to pollinators (the tepals or petals) have conical or papillate epidermal cells, at least on the adaxial surface (Kay *et al.*, 1981; Christensen and Hansen, 1998; Ojeda *et al.*, 2009). The observation that conical cells are widespread on petals but rare on leaves indicates that they function to increase floral attractiveness and plant reproductive success, a hypothesis that is supported by field trials in which wild-type flowers of *Antirrhinum majus* with conical cells received more insect attention and set more fruit than otherwise isogenic *mixta* mutant flowers with flat cells (Glover and Martin, 1998). Conical cell shape not only focuses light into petals, enhancing the pigmented colour (Kay *et al.*, 1981; Gorton and Vogelmann, 1996; Dyer *et al.*, 2007), but also provides a tactile advantage; bees can recognize different epidermal surfaces based on touch alone (Kevan and Lanet, 1985; Whitney *et al.*, 2009). Conical cells enable insects to minimize energy expenditure by ceasing wing movement and coming to rest while they feed, especially in natural conditions, when flowers are rarely stationary (Rands *et al.*, 2011; Alcorn *et al.*, 2012).

**Fig. 1.**
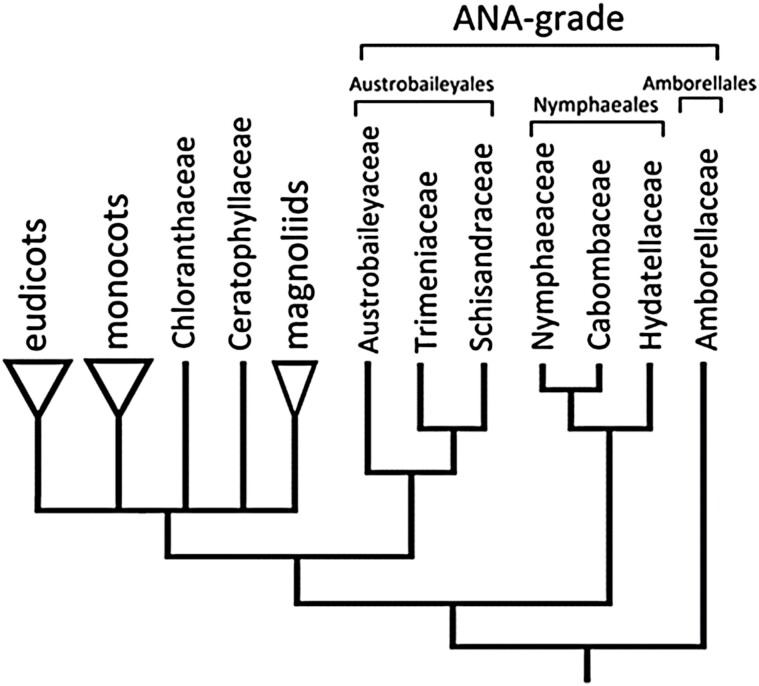
Relationships of the major angiosperm clades (Angiosperm Phylogeny Group, 2009), highlighting the ANA-grade lineages analysed in this study.

Conical tepal epidermal cells have been described across a phylogenetically broad range of angiosperm species, including many orders of eudicots and monocots (Kay *et al.*, 1981; Christensen and Hansen, 1998; Ojeda *et al.*, 2009; Taneda *et al.*, 2015). In contrast, relatively little is known about their distribution among the ANA-grade lineages (Amborellales, Nymphaeales, Austrobaileyales), which represent separate successive sister lineages to all other extant angiosperms in recent phylogenetic analyses (Fig. 1) (Angiosperm Phylogeny Group, 2009). The seminal investigation of Kay *et al.* (1981) did not include any ANA-grade families. Despite many morphological studies of flowers of ANA-grade species (e.g. Endress, 2008, 2010), existing descriptions of tepal surfaces are rare, with a few notable exceptions in the waterlily families Nymphaeaceae (Warner *et al*., 2008, 2009; Zini *et al.*, 2017; Coiro and Barone Lumaga, 2018) and Cabombaceae (Vialette-Guiraud *et al.*, 2011). To address these gaps, we explore the distribution of tepal epidermal cell morphologies across the ANA-grade orders.

The high morphological diversity among the relatively species-poor ANA-grade lineages, coupled with the absence of a extant outgroup closely related to angiosperms, make it difficult to reconstruct hypothetical ancestral states. However, extending information on both comparative morphology and gene function to the ANA-grade lineages is essential in understanding early angiosperm evolution. To explore these aspects, we analysed *MIXTA*-like gene function from an ANA-grade species that possesses conical cells: *Cabomba caroliniana* (Nymphaeales). In *A. majus*, loss of activity of the *MIXTA* gene leads to loss of the conical cell phenotype (Noda *et al.*, 1994), while ectopic expression of *MIXTA* in *Nicotiana tabacum* is sufficient to promote conical cell outgrowth (Glover *et al.*, 1998). MIXTA is a member of the R2R3 MYB transcription factor family, a subset of the MYB transcription factor family that is unique to plants (Martin and Paz-Ares, 1997; Dubos *et al.*, 2010). The R2R3 MYB transcription factors comprise 22 subgroups (Stracke *et al.*, 2001), with MIXTA falling into subgroup 9 (SBG9). Since the identification of *AmMIXTA*, three other *SBG9* genes have been identified in *A. majus*: *MYB MIXTA*-like (*AmMYBML*) *1*, *2*, and *3*, with overlapping but non-redundant functions (Perez-Rodriguez *et al.*, 2005; Baumann *et al.*, 2007; Jaffé *et al.*, 2007).

Phylogenetic analysis of SBG9 R2R3 *MYB* genes has revealed an ancient duplication that occurred before the origin of seed plants, resulting in two strongly supported clades: SBG9A, which encompasses *AmMIXTA* and *AmMYBML1*, *2*, and *3*), and SBG9B (Brockington *et al.*, 2013). SBG9A *MYB* genes have now been functionally characterized from the basal eudicot *Thalictrum thalictroides* (Di Stilio *et al.*, 2009), the monocot *Dendrobium crumenatum* (Gilding and Marks, 2010), and a range of eudicot species (Baumann *et al.*, 2007; Machado *et al.*, 2009; Gilding and Marks, 2010; Brockington *et al.*, 2013). To date, the only SBG9B gene that has been characterized is *MYB17-like* from *Lotus japonicus* (Brockington *et al.*, 2013). All SBG9 MYB transcription factors analysed so far play a role in epidermal cell outgrowth, often associated with petal conical cells.

The morphological data that we present here, exploring conical tepal epidermal cell distribution across the ANA-grade angiosperms, combined with our analysis of SBG9 MYB function in *C. caroliniana*, suggest that conical petal epidermal cells, and the anisotropic cell expansion that underpins their development in eudicots, are an ancestral feature of flowering plants.

## Materials and methods

### Sources of plant material

Materials of ANA-grade angiosperms examined for morphology are indicated in Table 1.

**Table 1.** Species and material examined

Family	Species examined	Material examined^*a*^
Amborellaceae	*Amborella trichopoda* Baill.	HK: *s.n.*
Nymphaeaceae	*N. odorata* Aiton subsp*. Tuberosa* Wiersema & Hellq.	HK: 1969-19765
	*N. violacea* Lehm.	HK: 2007-1810
	*Victoria cruziana* Orbign.	HK: 2011-1436
Cabombaceae	*C. caroliniana* A.Gray	HK: *s.n.* and commercial source (living plants)
	*C. furcata* Schult. & Schult.f	Commercial source (living plants)
Austrobaileyaceae	*Austrobaileya scandens* C.T.White	HK: 2012-464
Schisandraceae	*Kadsura heteroclita* Craib.	HK: 1985-4488
	*K. japonica* Benth.	HK: 1989-3952
	*Schisandra rubriflora* Rehder & E.H.Wilson	HK: 1969-19804
Illiciaceae	*Illicium floridanum* J.Ellis	HK: 2011-880
	*I. simonsii* Maxim.	HK: 1994-3682
Trimeniaceae	*Trimenia moorei* (Oliv.) Philipson	*s.n.*

^*a*^ HK indicates material cultivated at RBG Kew; *s.n.* indicates that the accession number is absent.

All *C. caroliniana* flowers and vegetative tissues for RNA/DNA extraction were purchased online from Plants Alive Ltd (Stone, Staffordshire, UK) and grown in a glass aquarium in 10 × 3.5 cm round pots with rockwool, weighted down using lead strips.

### Material preparation and preservation

Flowers and inflorescences were harvested and immediately fixed in FAA [60% ethanol (EtOH); 6% formaldehyde; 5% acetic acid]. The flowers were left in fixative for 72 h and then transferred to 70% EtOH solution. Where it was impractical to collect fresh material, dental wax (Elite HD vinylpolysiloxane) was used to make a high resolution mould of plant surfaces, and accurate replicas were produced using Devcon 2 Ton epoxy resin.

### Scanning electron microscopy

Prior to examination using SEM, fixed samples (stored in 70% EtOH) were dehydrated in a series of ascending EtOH concentrations and critical point dried in an Autosamdri 815B critical point drier. Samples were sputter-coated in platinum using an Emitech K550 sputter coater. The coated specimens were viewed using a Hitachi FE-SEM S-4700 scanning electron microscope (Hitachi Hi-Tec Technologies, Maidenhead, UK) and images captured using PCI software (Quartz Imaging Corp., Vancouver, Canada).

Epoxy cast material was coated in gold or chromium using a Quorum K756X sputter coater. Samples were then viewed using a FEI Philips XL30 FEGSEM scanning electron microscope 0.5–30 KeV with an Oxford Instruments INCA EDX system running a 30 mm^2^ SiLi thin window pentafet EDX detector.

### Isolation of *CcSBG9A1*

RNA was extracted from floral tissue frozen in liquid nitrogen, using a cetyltrimethylammonium bromide (CTAB)-based extraction followed by chloroform extraction and precipitation in 4 M LiCl. Prior to cDNA synthesis, RNA samples were treated with DNase I. cDNA was synthesized from total RNA using Bioline Bioscript™ reverse transcriptase, and oligo(dT) priming.

SBG9 R2R3 *MYB*-like sequences from ANA-grade angiosperms (*Amborella trichopoda* and *Nuphar* sp.) were obtained from the genomics database of the National Center for Biotechnology Information (NCBI; http://www.ncbi.nlm.nih.gov/). Sequences were aligned using Se-Al v2.0a11 and used to design degenerate primers. RACE was used to amplify full-length cDNAs which were cloned into pGEM-T for sequencing and further analysis. Primer sequences are listed in Supplementary Table S1.

### Phylogenetic analysis

The putative *Cabomba* SBG9A protein was analysed in the context of previously published alignments generated for the SBG9A clade (Brockington *et al.*, 2013). The sequence was aligned using the translation align function of nucleotide sequences in Geneious, and subject to a FastTree algorithm, using the GTR model. SH support values were generated during the FastTree analysis, and reported on the tree topology. All branches with <0.50 SH support were removed.

### Ectopic expression in *Nicotiana tabacum*

The binary vector pGREENII0029:35S with the *LacZ* gene removed and replaced with a double copy of the *Cauliflower mosaic virus* (CaMV) 35S gene promoter (Hellens *et al.*, 2000) was used for gene transfer. *CcSBG9A1* was inserted as an *Eco*RI fragment from pGEM-T into pGREEN. The binary vector was transferred into *Agrobacterium tumefaciens* GV3101 by electroporation. Transformation of *N. tabacum* var. Samsun was conducted using a modified version of the leaf disk protocol of Horsch *et al.* (1985). Transgenic plants were grown to maturity in a controlled greenhouse environment at 26 °C with a 16 h light regime, and transgene insertion and expression were confirmed using PCR with genomic DNA and reverse transcription–PCR (RT–PCR) with leaf RNA (see Supplementary Fig. S1).

### Quantitative RT–PCR (qPCR)

Mature flowers and buds were dissected on three independent plants using micro-dissecting forceps. Carpels, stamens, and tepals were removed separately and immediately frozen in liquid nitrogen. Forceps were cleaned with 100% ethanol between each tissue and flower. RNA for qPCR was extracted using Plant RNA Reagent (Invitrogen™), treated with Ambion TURBO DNA-free™, and converted to cDNA using Invitrogen Superscript III, primed using oligo(dT)_20_ and a random hexamer. *CcActin* was selected as a reference gene based on successful preliminary trials demonstrating stable expression across tissues. Primer sequences are provided in Supplementary Table S1. Forty cycles of PCR were performed using either a BioRad DNA Engine Thermocycler or a CFX Connect Real-Time PCR Detections System (185-5200). A melting curve was performed from 60 °C to 95 °C with readings taken at 0.5 °C intervals. Relative gene expression was quantified using an Opticon Monitor 3 and CFX Manager software (both BioRad Laboratories, Inc.). Ct values were exported to Microsoft Excel, and ∆Ct values were calculated by subtracting the Ct of the reference gene, actin. Each dataset was statistically analysed in Excel using a *t*-test.

## Results

### Conical or subconical tepal epidermal cells occur in several ANA-grade species

**Fig. 2.**
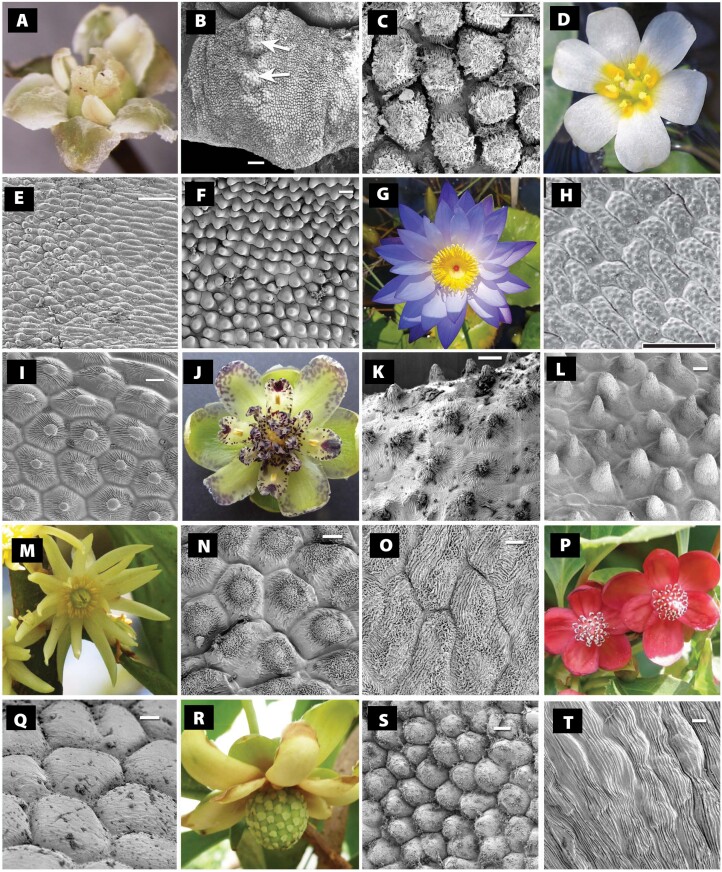
Adaxial epidermal morphology of coloured (insect-attracting) organs in ANA-grade flowers (photos and SEMs). (A–C) *Amborella trichopoda*, entire tepal showing central bulbous regions (arrowed) in (B) and detail of tepal surface showing domed flat-topped cells with chaotic surface patterning in (C). (D, E) *Cabomba caroliniana*, tepal surface at junction between flat-celled base (right) and mid-region with conical cells (left) in (E). (F) Detail of tepal surface of *Cabomba furcata* with conical cells. (G, H) *Nymphaea violacea*, detail of inner tepal surface in (H). (I) *Victoria cruziana*, detail of inner tepal surface showing cells with central prominence and radiating striations. (J–L) *Austrobaileya scandens*, details of inner tepal surface near margin (K), and staminode surface (L), both showing conical cells with fine striations. (M–O) *Illicium simonsii*, details of (N) mid-region of tepal showing cells with central prominence and chaotic striations, and (O) base of tepal showing flat cells with chaotic striations. (P, Q) *Schisandra rubriflora*, detail of tepal surface showing flat cells in (Q). (R, S) *Kadsura heteroclita*, detail of tepal surface showing domed cells in (S). (T) *Trimenia moorei*, detail of tepal surface showing flat cells. Scale bars=20 µm, except in (E)=100 µm, in (H)=50 µm, and in (I) and (Q)=10 µm.

A summary of tepal surfaces of ANA-grade species is given in Table 2 and Fig. 2, with emphasis on the distribution of conical cells and surface patterning. Following the terminology outlined by Kay *et al.* (1981), conical cells and papillate cells are more or less synonymous; they protrude significantly outwards from the epidermis and have a distinct tip or peak (subconical cells slightly less so). Lenticular cells are only slightly domed and lack a distinct tip. Flat cells show no clear sign of protrusion. In surface view, cell shape ranges from rounded to elongated, often on the same petal, with elongated cells mostly occurring at the petal/tepal base. In transverse section, cell shape ranges from flat through domed/lenticular to conical; as noted above, the presence of a distinct tip or peak separates conical cells from domed cells. Fine details of surface sculpturing range from smooth to striate.

**Table 2.** Morphology of the adaxial epidermis of tepals in ANA-grade families

Species	Tepal surface morphology	Data source
**Amborellaceae**		
*Amborella trichopoda*	Distinct conical cells absent, but cells strongly domed (subconical) and either smooth or with a flattened tip with fine surface ridges (Fig. 1A–C).	This study
**Cabombaceae**		
*Brasenia schreberi*	Conical cells absent; cells mostly flat and surfaces smooth; occasional long trichomes present.	Warner *et al.* (2008)
*Cabomba caroliniana*; *C. furcata*	Distinct conical cells present except at tepal bases, where cells are mostly flat (Fig. 1D–F).	This study
**Hydatellaceae**		
*Trithuria* spp.	Conical cells absent; cells flat and surfaces smooth.	Rudall *et al*. (2007, 2009)
**Nymphaeaceae**		
*Euryale ferox*	Conical cells absent; cells flat or slightly domed.	Coiro and Barone Lumaga (2018)
*Nymphaea caerulea; N. odorata* subsp. *tuberosa*; *N. violacea; N.* spp.	Conical cells absent; cells flat or slightly domed. Cell surfaces smooth (*N. odorata* subsp. *tuberosa*) or with numerous small lumps (micropapillae) on each cell (*N. violacea*, Fig. 1G, H).	This study; Warner *et al*. (2008, 2009); Coiro and Barone Lumaga (2018)
*Nuphar lutea*	Conical cells absent; cells flat and smooth.	Warner *et al*., 2008, 2009); Coiro and Barone Lumaga (2018)
*Victoria cruziana*	Conical cells absent from outer tepals but present on the inner staminoid tepals, where they possess a pronounced bulb at the tip, surrounded by radiating striations (Fig. 1I). Surfaces of other tepals with cells domed and smooth.	This study; Coiro and Barone Lumaga (2018)
**Austrobaileyaceae**		
*Austrobaileya scandens*	Conical cells present on most flower parts (Fig. 1J–L), especially the inner tepals, stamens, and staminodia, always with fine radiating striations. Outer tepals with relatively flat cells, except towards margins, where shallow conical cells are present.	This study
**Illiciaceae**		
*Illicium simonsii*; *I. floridanum*	Distinctly conical cells absent, but cells range from shallowly domed at the tepal base to more strongly domed towards the tepal apex, especially in *I. simonsii*. Clear striations present on surfaces of most cells, either axially oriented or chaotic (Fig. 1M–O).	This study
**Schisandraceae**		
*Kadsura heteroclita*; *K. japonica*	Distinctly conical cells occasionally present; cells ranging from flat to strongly domed or subconical, sometimes with an inflated tip (Fig. 1R, S). Surfaces relatively smooth or with fine nanoridges.	This study
*Schisandra rubriflora*	Conical cells absent; cells mostly flat or slightly domed (Fig. 1P, Q). Surfaces with an irregular pattern of shallow cuticular nanoridges.	This study
**Trimeniaceae**		
*Trimenia moorei*	Conical cells absent; cells mostly flat or slightly domed (Fig. 1T). Surfaces smooth or with axially oriented cuticular striations present. Central region of abaxial epidermis with long unicellular trichomes.	This study

ANA-grade taxa display a diverse tepal surface structure, consistent with their diverse floral morphology. Of the seven ANA-grade families, distinctly conical epidermal cells are present in species of *Austrobaileya* (Austrobaileyaceae), *Cabomba* (Cabombaceae), and the staminoid tepals of *Victoria cruziana* (Nymphaeaceae). In *Austrobaileya scandens*, conical cells cover most parts of the flower (tepals, stamens, and staminodia) except the carpels and the central regions of the outer tepals; all cells and papillae possess fine radiating striations. The adaxial tepal surface of *A. scandens* is complex, with large stomata and secretory cells also present; stomata are more abundant in central flat-celled regions that lack conical papillae. In *Cabomba*, non-striated conical cells cover the adaxial tepal surfaces of anthetic flowers, especially towards the tepal apex, with relatively flat surfaces at the tepal bases. In *V. cruziana*, only the innermost tepals and staminoid tepals have conical cells, which are often striated.

Subconical or deeply domed cells are present in *Amborella* (Amborellaceae), *Illicium* (Illiciaceae), and *Kadsura* (Schisandraceae). In *A. trichopoda*, the tepals are thick and reflexed, with a central adaxial groove surrounded by bulbous regions. The adaxial tepal surfaces display diverse morphology, though epidermal cells are mostly deeply domed, often flat-topped or angular with chaotic fine surface patterning. In *Illicium simmonsii*, tepal surfaces range from flat-celled to domed, sometimes with a central prominence and always with striations. In *Kadsura heteroclita*, most of the tepal surface is covered by subconical or occasionally conical cells. Conical and subconical cells are absent from Hydatellaceae, most Nymphaeaceae, *Schisandra* (Schisandraceae), and Trimeniaceae

### Regulation of conical epidermal cell growth in *Cabomba caroliniana*

#### Sequence and phylogenetic placement of *CcSBG9A1*

**Fig. 3.**
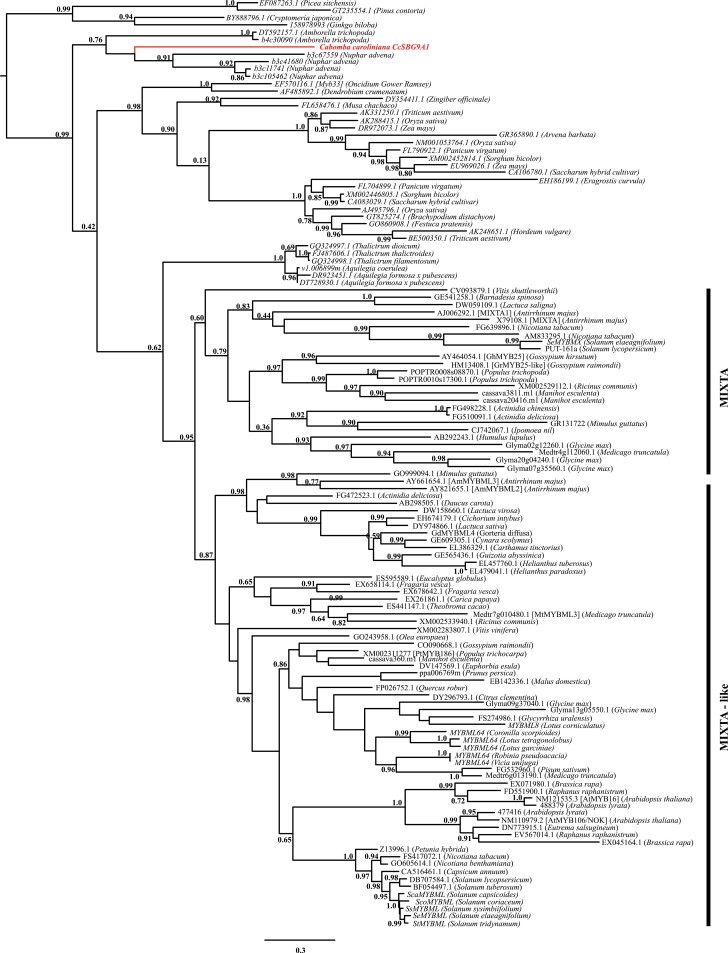
Maximum likelihood phylogram of SBG9A *MYB* genes from seed plants. SH support values are reported on the tree topology. The MIXTA and MIXTA-like clades of eudicot family members are marked. *CcSBG9A1* is highlighted in red.

To determine whether the anisotropic outgrowth of conical cells of ANA-grade tepals is regulated by the same R2R3MYB transcription factors (subgroup 9 MIXTA-like proteins) that control conical petal cell development in angiosperms, we isolated an SBG9A R2R3 *MYB* gene from *C. caroliniana* using degenerate PCR. The predicted protein contains the amino acid motif that is characteristic of the SBG9A lineage, which includes the well-characterized *MIXTA* and *MIXTA-*like genes from eudicots (Brockington *et al.*, 2013). The CcSBG9A1 protein shows a high degree of similarity with SBG9A MYB proteins from other ANA-grade genera. One notable exception is the occurrence of an amino acid substitution (lysine in place of threonine) at the centre of the highly conserved SBG9A motif. Phylogenetic analysis of SBG9A MYB genes, with the inclusion of the *CcSBG9A1* gene isolated here, confirms that *CcSBG9A1* groups with SBG9A MYB genes from other ANA-grade genera (*Nuphar* and *Amborella*) (Fig. 3; Supplementary Fig. S2). Together with monocot sequences and early diverging eudicot sequences, these ANA-grade sequences diverged before the main duplication within the core eudicots that gave rise to the *MIXTA* and *MIXTA-*like clades (Brockington *et al.*, 2013). While there are recent, lineage-specific duplications of this gene family within *A. trichopoda* and *Nuphar advena*, there is no evidence from this analysis of a deep duplication event within the ANA grade. Since our degenerate PCR identified no other gene fragments, there is only a single SBG9A EST from *Cabomba aquatica*, and our phylogenetic analysis provides no evidence of a deep duplication event, we tentatively conclude that there is only a single representative of MYB SBG9A in the *Cabomba* genome.

#### Transgenic analysis of CcSBG9A1 function in a tobacco bioassay

**Fig. 4.**
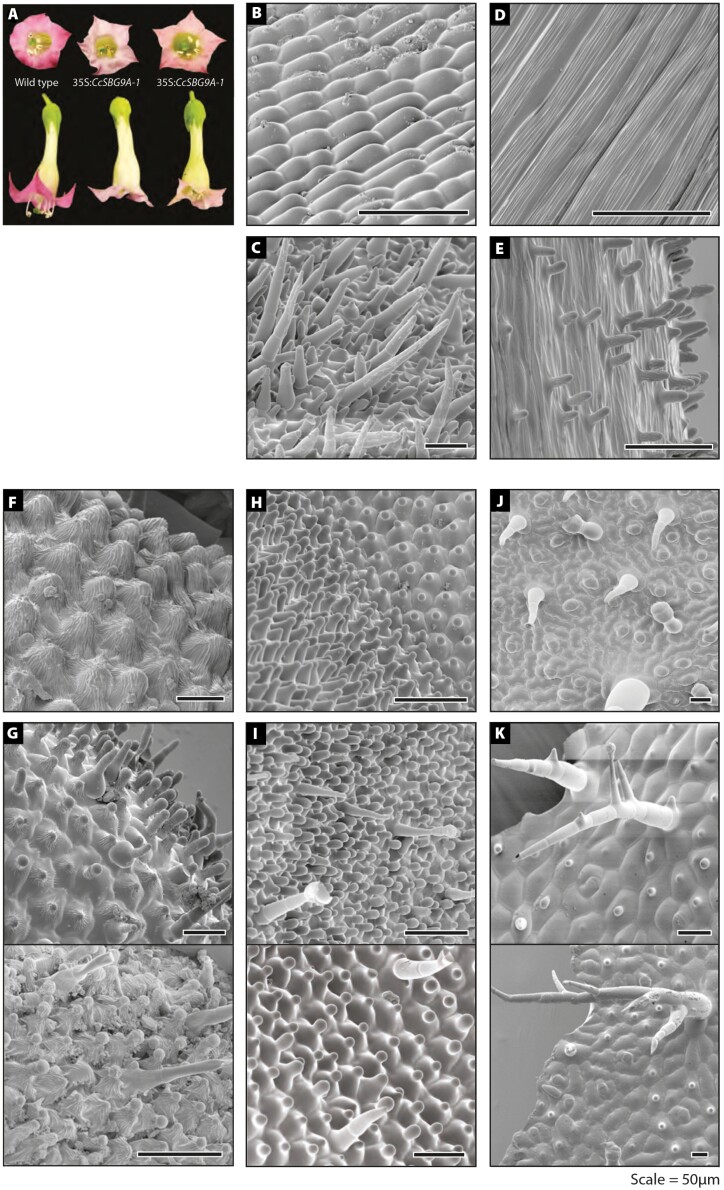
Ectopic expression of *CcSBG9A1* in tobacco. (A) Wild-type (left) and transgenic (centre, right) flowers. (B) SEM image of wild-type tobacco carpel epidermis. (C) SEM image of tobacco carpel expressing *CcSBG9A1.* (D) SEM image of wild-type tobacco style epidermis. (E) SEM image of tobacco style expressing *CcSBG9A1.* (F) SEM image of wild-type tobacco anther head. (G) SEM images of anther heads of two independent tobacco lines expressing *CcSBG9A1*. (H) SEM image of wild-type tobacco petal epidermis. (I) SEM images of petals of two independent tobacco lines expressing *CcSBG9A1*. (J) SEM image of wild-type tobacco leaf adaxial epidermis. (K) SEM images of adaxial leaf epidermis of two independent tobacco lines expressing *CcSBG9A1*. All scale bars=50 µm.

To explore the ability of the CcSBG9A1 protein to induce anisotropic cell expansion and cellular outgrowth, we generated nine independent transgenic lines of tobacco (*N. tabacum* var. Samsun) expressing the gene from the double CaMV35S promoter (Supplementary Fig. S1). The same bioassay has been used to explore the function of eudicot members of this gene family with different genes able to induce cellular outgrowth on different subsets of tobacco organs (Glover *et al.*, 1998; Perez-Rodriguez *et al.*, 2005; Baumann *et al.*, 2007; Jaffé *et al.*, 2007; Brockington *et al.*, 2013). The transgenic plants displayed a reduction in flower colour, the transgenic flowers appearing a much paler shade of pink relative to wild-type lines (Fig. 4A). The anthers of several of these lines—those also showing the strongest change in epidermal phenotype—failed to fully dehisce. These phenotypic outcomes have been described in other studies expressing SBG9A *MYB* genes in tobacco (Glover *et al.*, 1998).

Previous studies have reported that the ectopic expression of SBG9A *MYB* genes in tobacco most commonly induces cell outgrowth on the ovary epidermis. In wild-type plants, epidermal cells of the ovary have a rounded base shape and are flat or slightly lenticular (Fig. 4B). In all lines of transgenic tobacco expressing 35S:*CcSBG9A1*, ectopic cell outgrowths were present on the surface of the ovary (Fig. 4C). The majority of epidermal cells had an altered appearance, and conical cells and trichomes were present in approximately equal abundance. These cell protrusions ranged from 10 µm to 350 µm in length.

In wild-type tobacco flowers, the style and stamen filaments have uniformly flat elongate cells (Fig. 4D). In transgenic lines expressing 35S:*CcSBG9A1*, ectopic cell protrusions were observed on both floral organs, although they were less dense than on the ovary. On the style of plants with a strong phenotype, protrusions ranged from conical cells to long-stalked trichomes (Fig. 4E). On the stamen filament of transgenic flowers, short trichomes <30 µm in length were the most common type of protrusion. The anther of wild-type flowers has a regular arrangement of conical cells (Fig. 4F). In all transgenic lines, epidermal cells had an altered shape and distinct bulbous tip (Fig. 4G). For some cells, the tips of cells were extended into trichomes of varying lengths.

At the tip of the corolla, the epidermis of wild-type flowers has a regular arrangement of conical cells with a pronounced bulb at the tip of each cell (Fig. 4H). In all lines expressing 35S:*CcSBG9A1*, epidermal cell shape was more variable particularly at the tips of cells, which protruded to varying degrees (Fig. 4I). Multicellular trichomes >50 µm in length, and sometimes glandular, were found to be sparsely distributed amongst the conical cells.

On the adaxial epidermis of wild-type leaves, cells are largely flat with a rounded base shape. Long multicellular trichomes and shorter hydathode-type trichomes are irregularly distributed on the leaf epidermis (Fig. 4J). In several transgenic lines, some of the long multicellular trichomes had multiple branches (Fig. 4K). Between these trichomes, the epidermal cells remained largely flat or lenticular, but many of these cells developed a distinct peak or tip (Fig. 4K). Occasionally, these cells also had an altered overall shape and were distinctly conical. No changes in epidermal morphology were observed on the abaxial leaf epidermis of transgenic lines.

### Ontogeny of tepal epidermal outgrowth in *Cabomba caroliniana*

**Fig. 5.**
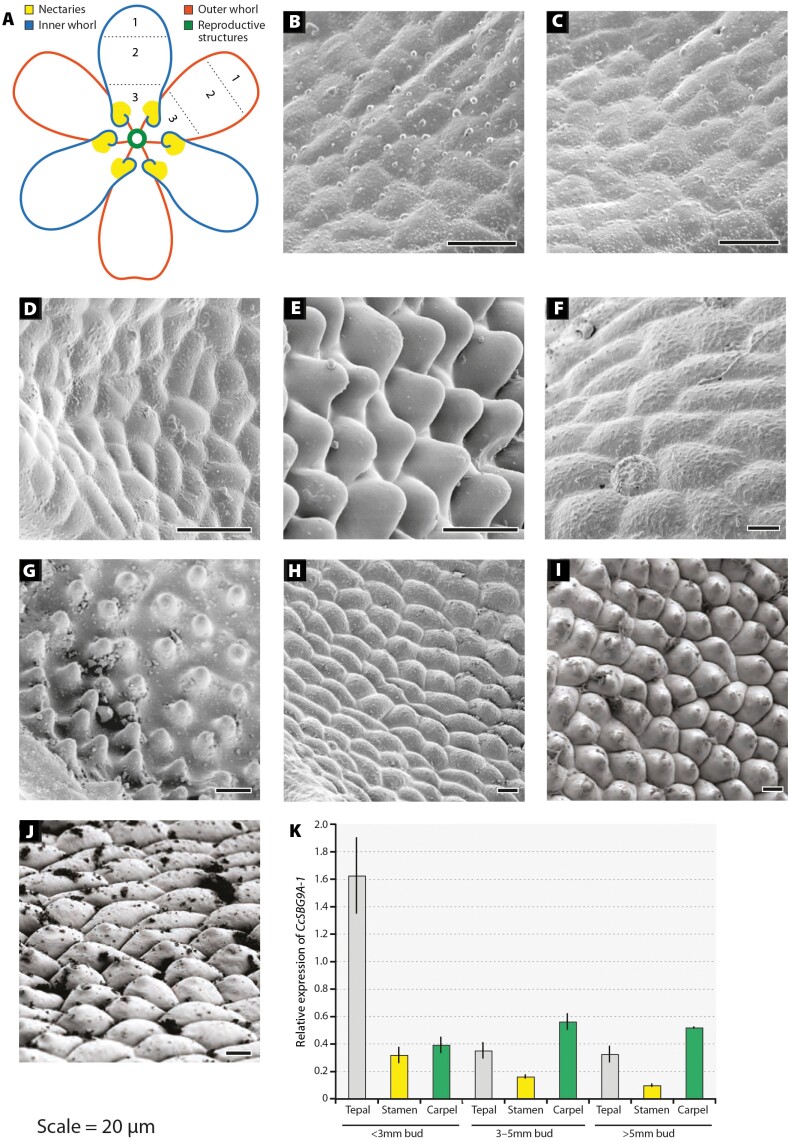
Development of conical tepal epidermal cells in *Cabomba caroliniana.* (A) Schematic diagram showing the tepal morphology of *Cabomba* as used for the SEM developmental series. Carpels and stamens are not shown. The positions of the inner and outer tepal whorls (see key), nectaries, and sampling zones (1–3) are marked. (B) SEM image of adaxial tepal epidermis at stage 1, zone 3. Inner and outer tepals are indistinguishable at this stage. (C) SEM image of adaxial epidermis of inner tepal at stage 2, zone 3. (D) SEM image of adaxial epidermis of outer tepal at stage 2, zone 1. (E) SEM image of adaxial epidermis of inner tepal at stage 3, zone 1. (F) SEM image of adaxial epidermis of outer tepal at stage 3, zone 3. (G) SEM image of adaxial epidermis of inner tepal at stage 4, zone 1. (H) SEM image of adaxial epidermis of outer tepal at stage 4, zone 1. (I) SEM image of adaxial epidermis of inner tepal at stage 5, zone 1. (J) SEM image of adaxial epidermis of outer tepal at stage 5, zone 1. (K) qPCR analysis of *CcSBG9A-1* expression in different tissues and at different developmental stages (<3 mm=stages 1 + 2; 3–5 mm=stages 3 + 4; >5 mm=stage 5;) of *Cabomba caroliniana*. Target gene expression was quantified relative to actin. Values represent mean expression values and SEs (*n*=3). All scale bars=20 µm.

Tepal epidermal morphology was characterized at five developmental stages of *C. caroliniana*: (1) 1 mm buds; (2) 2 mm buds; (3) 4 mm buds; (4) 5 mm buds; and (5) 7 mm buds or flowers at anthesis. Flowers of *Cabomba* have whorled floral phyllotaxy, with two whorls of three petaloid tepals forming in alternating positions (Fig. 5A). The inner tepals are developmentally retarded with respect to the outer tepals and other floral organs, and thus for each stage the tepals from the inner and outer whorls were imaged separately (Vialette-Guiraud *et al.*, 2011). Specific zones along the length of the tepal were identified for comparative analysis, as outlined in Fig. 5A.

At the youngest stage (stage 1), the inner and outer whorls of tepals were indistinguishable, and there was no evidence of cell outgrowth (Fig. 5B). By stage 2, nectaries are present towards the base of the inner tepals, although these are restricted to the very outer edges of the tepal (Fig. 5C). Cells at the tip of the tepal are similar in appearance in inner and outer tepal whorls (Fig. 5D), while those at the base of the tepal are more variable. There was no evidence of cell outgrowth. By stage 3, pronounced conical cells are visible at the tip of both the inner and outer tepals. On the inner tepals, these cones have a more pointed shape (Fig. 5E), while they are distinctly rounded on the outer whorl. At the base of the inner tepal, nectaries are well developed (Fig. 5F). Stage 4 shares an almost identical phenotype with stage 3 (Fig. 5G, H). By stage 5, conical epidermal cells at the tips of the tepals are more uniform, but there is no change in their total degree of protrusion (Fig. 5I, J).

### Expression analyses of *CcSBG9A1* in *Cabomba caroliniana*

qPCR was used to determine whether expression levels of *CcSBG9A1* correlated with conical cell development in *C. caroliniana*. Expression analyses were conducted across a range of floral tissues—tepals (pooled inner and outer whorls), stamens, and carpels—and three developmental stages [pooled bud stages 1 and 2 (<3 mm), pooled bud stages 3 and 4 (3–5 mm in length), and bud stage 5 (>5 mm in length)]. Mean expression values for each tissue and developmental stage were calculated relative to expression levels of *CcActin* from three technical and three biological replicates (Fig. 5K). The highest level of *CcSBG9A1* expression was in young tepals at stages 1 and 2, immediately prior to the appearance of conical cells (at stage 3). There were very low levels of *CcSBG9A1* expression in tepals larger than 3 mm and in mature flowers (>5 mm). A *t*-test confirmed that *CcSBG9A1* expression in the tepals of <3 mm buds is significantly higher than in the tepals of >5 mm buds [*t*(4)=5.92, *P*<0.01]. When *CcSBG9A1* expression is compared in the different tissues of <3 mm buds, it is significantly higher in the tepals relative to the stamens [*t*(4)=6.37, *P*<0.01] or carpels [*t*(4)=6.12, *P*<0.01].

## Discussion

### Conical tepal epidermal cells are present in several ANA-grade angiosperms

Our examination of species from the three ANA-grade orders (Fig. 2) reveals complex tepal surfaces in ANA-grade species, consistent with the diversity of flower structure in these taxa which contributes to the bigger picture of perianth epidermal morphology evolution across the angiosperms. Tepal surfaces are rarely entirely uniform, and can differ on the same flower and even on the same tepal. Two ANA-grade genera, *Cabomba* and *Austrobaileya*, possess distinctly conical cells over most of the adaxial tepal surface. In several other ANA-grade genera (e.g. *Kadsura* and *Victoria*), cells are conical or subconical on some parts of the tepal surface. *Amborella trichopoda*, the putative sister to all other angiosperms, possesses strongly domed cells. In contrast, a few ANA-grade species possess mostly flat cells on the tepal surface (e.g. *Nuphar* and *Trimenia*).

The conical-papillate petal epidermis represents the most common type in angiosperms (Kay *et al.*, 1981), but there also exist many subconical types with a rounded or flattened apex, as we have found in *Amborella* and *Kadsura*. The widespread distribution of conical or subconical cells in all three ANA-grade lineages indicates that the capacity to produce them is of ancient origin. Few studies have examined the apparently simple transition from a lenticular or subconical cell to a conical cell. In many eudicots, formation of both conical cells and trichomes on the petal epidermis is determined by SBG9A MYB transcription factors (Martin *et al.*, 2002. Perez-Rodriguez *et al.*, 2005). Ectopic expression of SBG9A MYB genes can induce both conical and lenticular cellular outgrowth (Martin *et al.*, 2002; Jaffé *et al.*, 2007). In some eudicots, there is clear evidence for an evolutionary loss of the conical cell form within a specific taxonomic group or natural community (e.g. Ojeda *et al*., 2009, 2016).

Pollination biology is also diverse among ANA-grade species, though data are relatively sparse for some taxa (Thien *et al.*, 2009; Endress, 2010; Luo *et al.*, 2018). Beetle pollination is common in the waterlily family Nymphaeaceae and in some Schisandraceae; flies are the major pollinators of *Austrobaileya* and *Illicium*; and Schisandraceae are predominantly pollinated by nocturnal gall midges (Endress, 2010; Luo *et al.*, 2018). Petal surfaces with domed and/or conical cells are frequently involved in scent production (Vogel, 1990). The tiny white flowers of *Amborella* produce a scent that attracts nocturnal moths and other insects (Thien *et al.*, 2009). A likely source for the scent is the prominent regions of the central part of the tepal surface, which function as osmophores. The waterlily genus *Cabomba*, which possesses prominent conical cells, is unusual among ANA-grade angiosperms in possessing well-defined nectaries on the surfaces of the inner tepals; the nectar provides a reward to visiting pollinating insects such as bees, wasps, and flies (Schneider and Jeter, 1982; Taylor and Williams, 2009; Vialette-Guiraud *et al.*, 2011; Luo *et al.*, 2018). Two genera that lack conical cells are probably abiotically pollinated: *Trithuria* (Hydatellaceae) and *Brasenia* (Cabombaceae), supporting a correlation between conical cells and pollinator attraction.

### An SBG9A MYB transcription factor from an early diverging angiosperm can induce ectopic conical cell development

To analyse the homologies of conical tepal epidermal cells, we explored the developmental genetic processes underpinning cellular differentiation. A common developmental programme could suggest a single ancestral origin followed by repeated evolutionary losses or modifications. The SBG9A MYB transcription factors are known to control petal epidermal cell outgrowth in both eudicots (Noda *et al.*, 1994; Machado *et al.*, 2009; Di Stilio *et al.*, 2009; Brockington *et al.*, 2013) and monocots (Gilding and Marks, 2010). We therefore examined whether this subgroup of *MYB* genes could perform similar functions in ANA-grade angiosperms, suggesting a single origin of conical cells.

Analyses of SBG9A MYB protein function are sometimes hampered by the many duplications seen within the gene family at different phylogenetic levels (Bedon *et al.*, 2014). However, our phylogenetic reconstruction, coupled with evidence from published transcriptomes, demonstrates only a single SBG9A gene in the *Cabomba* genome. The gene family is divided into MIXTA and MIXTA-like clades following a duplication at the base of the eudicots (Brockington *et al.*, 2013), but the ANA-grade members form a clade that diverged before this duplication (Fig. 2). Furthermore, although there are lineage-specific duplications in some genera within this clade, we found no evidence for a deep duplication event within the ANA-grade lineages.

Ectopic expression of *CcSBG9A1* in tobacco revealed that the protein has the ability to induce anisotropic cell expansion and cellular outgrowth in all tissues tested, indicating that it is able to induce cellular differentiation alone (or with a ubiquitously expressed partner). The strength of the ectopic expression phenotype is remarkable. The same heterologous approach using the same strong constitutive promoter (a double copy of the 35S promoter from CaMV) in *N. tabacum* has been used for several other angiosperm SBG9A MYB genes over the last 20 years. This list includes *TtMYBML2* from the basal eudicot *Thalictrum thaloctroides* (Di Stilio *et al.*, 2009), *AtMYB16* and *AtMYB106* from Arabidopsis (Baumann *et al.*, 2007; Gilding and Marks, 2010), *PhMYB1* from *Petunia hybrida* (Baumann *et al.*, 2007), and the four SBG9-A genes in *A. majus* (*AmMIXTA*, *AmMIXTA-LIKE 1*, *AmMIXTA-LIKE2*, and *AmMIXTA-LIKE 3*) (Glover *et al.*, 1998; Perez-Rodriguez *et al.*, 2005; Baumann *et al.*, 2007; Jaffé *et al.*, 2007). It is notable that *CcSBG9A1* induces a much stronger phenotype than most previously characterized SBG9A MYB genes in this bioassay. For example, *PhMYB1*, *AmMYBML2*, *AtMYB16*, and *TtMYBML2* share similar expression patterns and similar phenotypes when ectopically expressed in tobacco. Transgenic tobacco plants exhibit ectopic outgrowths on the surface of the ovary, as well as an increase in the height and change in shape of conical cells on the corolla. However, these outgrowths never develop into multicellular trichomes, and no changes were observed to the other floral organs, or vegetative leaves (some changes were observed on inflorescence leaves) (Baumann *et al.*, 2007; Di Stilio *et al.*, 2009). The strongest reported phenotypes from this bioassay are for *N. tabacum* plants overexpressing *AmMIXTA*, which exhibit long multicellular trichomes on the ovary and at the tip of the inner corolla. On the leaves, several parallels can be drawn with the effects of *CcSBG9A1* expression. For example, the majority of cells on the adaxial leaf epidermis have a single, central outgrowth. These outgrowths are almost identical on the adaxial leaf epidermis of transgenic tobacco expressing 35S:*CcSBG9A1* and 35S:*AmMIXTA*. Long-stalked, multicellular branched trichomes were also observed on the adaxial leaf epidermis of both transgenic lines (Glover *et al.*, 1998; Perez-Rodriguez *et al.*, 2005). Phenotypic strength may be affected by the position of transgene insertion and the transgene expression level, so our conclusions here must be tentative, but it is nonetheless notable that the phenotypes observed in this study are consistently stronger than those for most related genes using the same bioassay system.

The four SBG9A genes in *A. majus* have arisen from Antirrhineae-specific duplication events within the MIXTA (*AmMIXTA* and *AmMIXTA-LIKE 1*) and MIXTA-like (*AmMIXTA-LIKE2* and *AmMIXTA-LIKE 3*) clades. These four genes show sequence homology and may have overlapping functions, but they are not functionally redundant. It has been suggested that formation of fully developed conical cells on the petals of *A. majus* requires two distinct activities. For example, *AmMIXTA* and *AmMYBML1* may be responsible for initiating conical cell development, while *AmMYBML2* and *AmMYBML3* coordinate a second stage of elongation that leads to a complete cone (Perez-Rodriguez *et al.*, 2005).

Our study shows that the gene duplication event that led to formation of the MIXTA and MIXTA-like clades, as well as the Antirrhineae-specific duplication event that gave rise to *AmMYBML1*, *2*, and *3*, arose after the divergence of *Cabomba* (Fig. 2). There is no evidence of an ancient gene duplication event in the MIXTA and MIXTA-like clades within either the early diverging angiosperm or the early land plant lineages. In turn, we infer that the single CcSBG9A1 protein plays a crucial role in inducing anisotropic cell expansion and coordinating conical cell development in *C. caroliniana*. The ability of *CcSBG9A1* to induce the formation of ectopic cell outgrowths in tobacco indicates that the encoded CcSBG9A1 protein is capable of regulating transcriptional targets similar to other members of the SBG9A lineage. The strength of the phenotype suggests that CcSBG9A1 is a particularly effective transcriptional regulator of the downstream cellular differentiation pathway and has the potential to act as a master regulator of epidermal cell outgrowth.

### A SBG9A MYB transcription factor is expressed specifically in developing tepals of *Cabomba caroliniana*, immediately prior to conical cell outgrowth

Since it is not possible to transform *C. caroliniana*, we sought additional correlative evidence in support of a role for *CCSBG9A1* in conical cell development. Although CcSBG9A1 is clearly able to induce cellular outgrowth, its native phenotypic effects will depend on the transcriptional profile of the gene encoding it. We used an ontogenetic series to determine that epidermal cell outgrowth in the *Cabomba* tepal occurs at growth stage 3, when buds are between 2 mm and 4 mm long. We predicted that the transcriptional regulator controlling cellular outgrowth would be expressed in earlier stages of tepal development. qPCR analyses of dissected tissues revealed that *CcSBG9A1* is most strongly expressed in tepals of buds <3 mm in length. Transcript is almost undetectable in other floral organs (stamens and carpels) and in tepals at later developmental stages when the conical cells are already present. This expression pattern correlates strongly with a role in regulating conical tepal epidermal cell development.

### Conclusions

Our study clearly demonstrates the presence of conical perianth epidermal cells in some of the earliest surviving angiosperm lineages. Our combined strong, if correlative, evidence suggests that outgrowth of the conical cells in *Cabomba* is regulated by the same MYB SBG9A-initiated pathway that regulates petal cell development in eudicots. This ancient origin for conical cells and their developmental programme suggest that the many angiosperm species that lack conical petal cells represent secondary losses of an ancestral character. We hypothesize that changes in *cis* regulation or protein function of *SBG9* MYB genes, potentially correlated with shifts in pollinator type and behaviour, are responsible for the repeated loss of conical cells in many lineages.

## Supplementary data

The following supplementary data are available at *JXB* online.

Fig. S1. Genotyping transgenic tobacco lines expressing *CcSBG9A-1*.

Fig. S2. Maximum likelihood phylogram of SBG9A *MYB* genes from seed plants.

Table S1. Primer sequences.

erac223_suppl_Supplementary_Materials

## Data Availability

The sequence of *CcSBG9A1* is deposited in the GenBank database under accession number ON364012. All materials are available on request from BJG.
