# Supplementary material for: Conical petal epidermal cells, regulated by the MYB transcription factor MIXTA, have an ancient origin within the angiosperms
Source: J Exp Bot. 2022 May 21;73(16):5490–502. doi: 10.1093/jxb/erac223 (PMC9467652; doi:10.1093/jxb/erac223)
Supplement: erac223_suppl_Supplementary_Materials [file erac223_suppl_supplementary_materials.pdf]

Supplementary Data

Article title: **Conical petal epidermal cells, regulated by the MYB transcription factor MIXTA, have an ancient origin within the angiosperms**

Authors: Alison Reed, Paula J. Rudall, Samuel F. Brockington and Beverley J. Glover

The following Supplementary Data are available for this article:

Fig. S1 Genotyping transgenic tobacco lines expressing *CcSBG9A-1*. A. Gel electrophoresis of PCR using genomic DNA extracted from 14 lines of transgenic tobacco. For each line two independent primer combinations were used. All 14 lines reveal the transgene with both primer combinations, while the wild type control (WT) produces no bands. B. Gel electrophoresis of RT-PCR to confirm expression of the *CcSBG9A-1* transgene in 9 independent transgenic lines. The pair of transgene specific primers amplify a band in all 9 lines, but not in the wild type (WT) control.

S1a

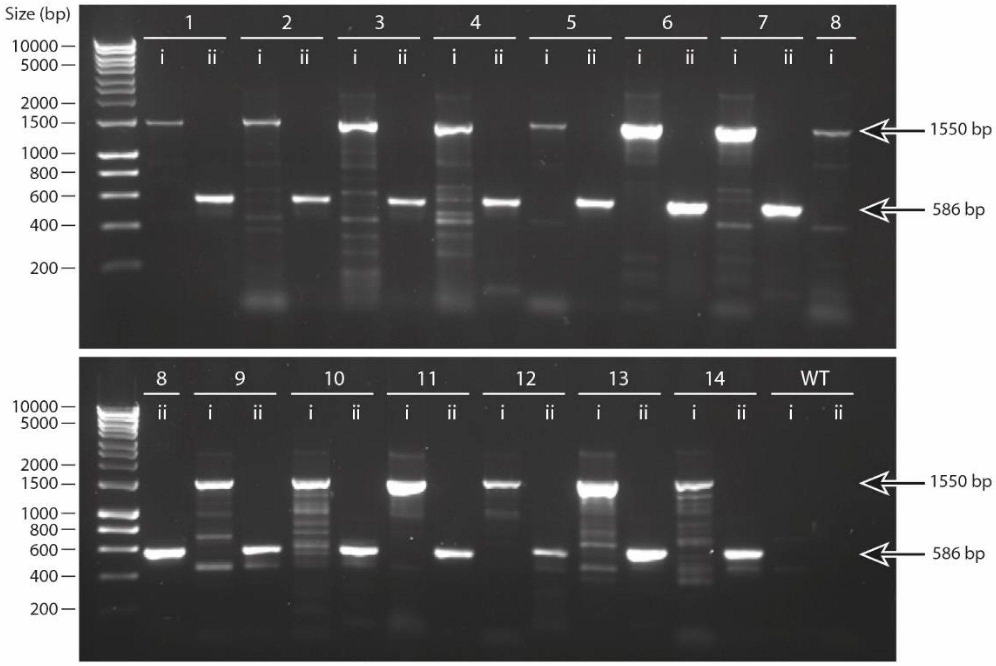
Size (bp)
10000
5000
2000
1500
1000
800
600
400
200
1 2 3 4 5 6 7 8
i ii i ii i ii i ii i ii i ii i ii i
8 9 10 11 12 13 14 WT
ii i ii i ii i ii i ii i ii i ii i ii
1550 bp
586 bp

S1b

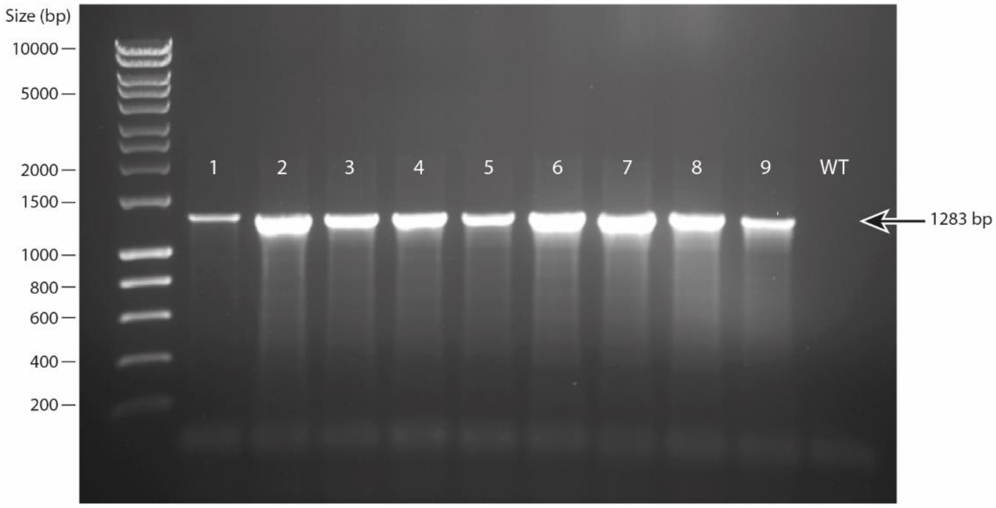
Size (bp)
10000
5000
2000
1500
1000
800
600
400
200
1 2 3 4 5 6 7 8 9 WT
1283 bp

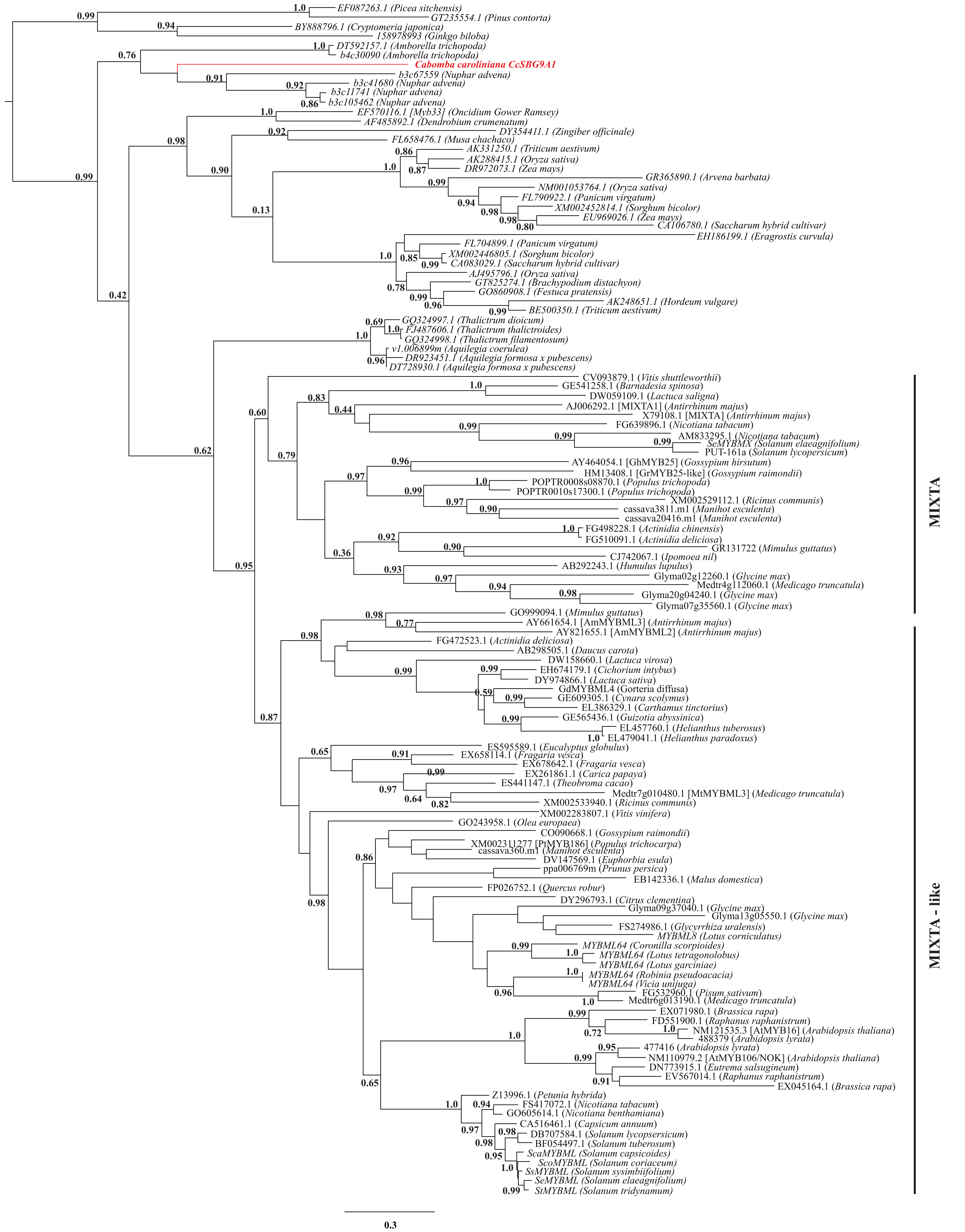

Figure S2. Maximum likelihood phylogram of SBG9A MYB genes from seed plants. SH support values are reported on the tree topology. The MIXTA and MIXTA-like clades of eudicot family members are marked. CcSBG9A1 is highlighted in red.

Table S1. Primer sequences

Primers to amplify CcSBG9A-1

CaboMIXTA_4F TTCAGATGGTCGGCCATCG (forward)

CaboMIXTA_5R TCTAGGCGGGCGCTCTCC (reverse)

CaboMIXTA_7F GCGGACGGACAATGAAATCAAAA (forward, to get 5’end)

Cabodeg9 ATGGGYCGRTCTCCTTGTTGTGASAAG (forward, to get start codon)

Cabospec14 TCAGAAAAAAGGAAAGGCCGC (reverse)

Primers for qPCR

qPCR_CaboMIXR GCTCCTCCTGCAGACTGAATT (reverse)

qPCR_CaboMIXF CCTATCAAAGCTGGTTTGCTCAGG (forward)

CabActqPCRF GGACGTACTACTGGTATTGTGATGG (actin, forward)

CabActqPCRR CATCAGCGAATCGGTCAGATCA (actin, reverse)
